# High spatial resolution free-breathing 3D late gadolinium enhancement cardiac magnetic resonance imaging in ischaemic and non-ischaemic cardiomyopathy: quantitative assessment of scar mass and image quality

**DOI:** 10.1007/s00330-018-5361-y

**Published:** 2018-04-06

**Authors:** Maurice B. Bizino, Qian Tao, Jacob Amersfoort, Hans-Marc J. Siebelink, Pieter J. van den Bogaard, Rob J. van der Geest, Hildo J. Lamb

**Affiliations:** 1 Department of Radiology, Leiden University Medical Center, Albinusdreef 2, 2333 ZA Leiden, The Netherlands; 2 Department of Cardiology, Leiden University Medical Center, Albinusdreef 2, 2333 ZA Leiden, The Netherlands

**Keywords:** Late gadolinium enhancement, MRI, High spatial resolution, Myocardial infarction, Non-ischaemic cardiomyopathy, Free-breathing

## Abstract

**Purpose:**

To compare breath-hold (BH) with navigated free-breathing (FB) 3D late gadolinium enhancement cardiac MRI (LGE-CMR)

**Materials and methods:**

Fifty-one patients were retrospectively included (34 ischaemic cardiomyopathy, 14 non-ischaemic cardiomyopathy, three discarded). BH and FB 3D phase sensitive inversion recovery sequences were performed at 3T. FB datasets were reformatted into normal resolution (FB-NR, 1.46x1.46x10mm) and high resolution (FB-HR, isotropic 0.91-mm voxels). Scar mass, scar edge sharpness (SES), SNR and CNR were compared using paired-samples *t*-test, Pearson correlation and Bland-Altman analysis.

**Results:**

Scar mass was similar in BH and FB-NR (mean ± SD: 15.5±18.0 g vs. 15.5±16.9 g, *p*=0.997), with good correlation (r=0.953), and no bias (mean difference ± SD: 0.00±5.47 g). FB-NR significantly overestimated scar mass compared with FB-HR (15.5±16.9 g vs 14.4±15.6 g; *p*=0.007). FB-NR and FB-HR correlated well (r=0.988), but Bland-Altman demonstrated systematic bias (1.15±2.84 g). SES was similar in BH and FB-NR (*p*=0.947), but significantly higher in FB-HR than FB-NR (*p*<0.01). SNR and CNR were lower in BH than FB-NR (*p*<0.01), and lower in FB-HR than FB-NR (*p*<0.01).

**Conclusion:**

Navigated free-breathing 3D LGE-CMR allows reliable scar mass quantification comparable to breath-hold. During free-breathing, spatial resolution can be increased resulting in improved sharpness and reduced scar mass.

**Key Points:**

• *Navigated free-breathing 3D late gadolinium enhancement is reliable for myocardial scar quantification.*

• *High-resolution 3D late gadolinium enhancement increases scar sharpness*

• *Ischaemic and non-ischaemic cardiomyopathy patients can be imaged using free-breathing LGE CMR.*

**Electronic supplementary material:**

The online version of this article (10.1007/s00330-018-5361-y) contains supplementary material, which is available to authorized users.

## Introduction

Late gadolinium enhancement (LGE) imaging is an established cardiovascular magnetic resonance (CMR) technique to assess myocardial viability [1]. LGE-CMR is commonly used in the diagnosis of ischaemic cardiomyopathy (ICM) and to guide revascularisation therapy. In addition, LGE-CMR has the ability to detect partially infarcted myocardial tissue, known as grey zone, which is believed to be a substrate for arrhythmia [2]. Grey zone mass quantification using LGE-CMR has been shown to predict post-myocardial infarction mortality [3]. In non-ischaemic cardiomyopathy (NICM), a specific LGE pattern allows for a differential diagnosis [1]. More recently, scar mass quantification has been shown to predict adverse cardiovascular outcome in various non-ischaemic cardiomyopathies [4–6]. The increasing range of clinical applications warrants optimisation of current LGE-CMR protocols.

Standard LGE-CMR is performed during multiple breath-holds in several pre-defined two-dimensional (2D) orientations with an inversion recovery (IR) gradient echo sequence with the inversion time manually chosen to null the signal of healthy myocardium [1]. Three-dimensional (3D) LGE-CMR is desirable because it allows (1) imaging of the whole heart without slice gaps, (2) imaging with high SNR and CNR, and (3) performing post-acquisition reformatting in any desired plane [7, 8]. However, 3D LGE-CMR is hampered by long breath-hold duration (>20 s) [9], thereby compromising its robustness in a clinical setting that includes vulnerable patients [10]. To overcome these limitations, 3D LGE-CMR can be performed during free-breathing, for example with respiratory gating based on diaphragmatic pencil-beam navigation [7]. The free-breathing approach allows to apply a phase sensitive inversion recovery sequence (PSIR) in combination with high spatial resolution (submillimeter voxel size). The PSIR sequence triggers inversions every other heartbeat with an additional proton density-weighted reference image acquired on alternate heartbeats. As a result, PSIR reconstructed images are less sensitive to wrong inversion time and heart rate variability as compared with standard inversion recovery [7]. In theory, high spatial resolution LGE-CMR should result in better defined scar borders (specified as scar edge sharpness, SES), which could diminish partial volume averaging that contributes to scar and grey zone mass overestimation [11, 12]. To date, breath-hold 3D LGE-CMR has not been compared with free-breathing 3D LGE-CMR with matched spatial resolution. Furthermore, high spatial resolution free-breathing 3D LGE-CMR has not been compared with normal spatial resolution free-breathing 3D LGE-CMR in a clinical setting.

Therefore, the purpose of this study was to compare scar mass, grey zone mass, SES, and image quality of late gadolinium enhancement cardiac MRI using multiple breath-hold 3D phase sensitive inversion recovery imaging with matched normal spatial resolution navigated free-breathing 3D LGE-CMR (FB-NR), and to compare FB-NR with high spatial resolution navigated free-breathing 3D LGE-CMR (FB-HR) at 3.0 Tesla in ischaemic and non-ischaemic cardiomyopathy patients.

## Methods

### Ethics

Navigated free-breathing LGE-CMR validation against standard breath-hold LGE-CMR was performed within the frame of clinical CMR protocol development. This retrospective study was considered a chart review. Therefore, written informed consent was waived by the hospital institutional review board.

### Patient population

From May 2012 until November 2013, breath-hold and free-breathing sequences were acquired in patients that were referred for LGE-CMR, if there was enough time in the clinical scanning slot. Of the 54 patients in which both BH and FB were performed, there were two incomplete free-breathing datasets (one acquisition was stopped due to allergic contrast reaction; one was stopped because of very low navigator efficiency). In one patient there was no LGE detectable. The remaining 51 patients were included (38 male; 13 female; mean age ± standard deviation: 59.9 years ± 11.4 years).

### MRI

All CMR examinations were performed in our institution on a 3T unit (Ingenia, Philips Healthcare, Best, The Netherlands) with the patient in supine position. The body coil was used for transmission, and a 16-element anterior and 12-element posterior phased-array coil were used for signal reception. Following a standard clinical CMR protocol including 2D cine imaging in all cardiac axis views, the gadolinium-based contrast agent gadoterate meglumine (Dotarem, Guerbet, Villepinte, France) was administered intravenously at a dose of 0.15 mmol/kg. Ten minutes after contrast administration, a 2D gradient-echo T1 weighted sequence was used (i.e. Look-Locker) to visually determine the optimal inversion time (TI) of healthy myocardium. Approximately 10–15 min post-contrast, a whole heart high spatial resolution 3D gradient echo (T1 fast field echo) PSIR sequence was acquired during free breathing with diaphragmatic pencil-beam navigation. Image parameters were as follows: repetition time (TR) 4.15 ms; echo time (TE) 2.02 ms; TI was set at the null point plus 50 ms and ranged from 250 to 400 ms; flip angle (FA) 10°; field of view (FOV) 350 × 350 mm; matrix size 208 × 208; acquired pixel size 1.68 × 1.68 mm; reconstructed pixel size 0.91 × 0.91 mm; 71 transverse slices with 3.4 mm thickness and slice gap -1.7 mm; sensitivity encoding (SENSE) factor 3. The 2D pencil beam respiratory navigator was planned on the right hemidiaphragm. The acceptance window was set at 5 mm with a constant correction of 0.6. Saturation bands were planned on the navigator, vertebrae and thoracic subcutaneous adipose tissue to suppress residual motion artefacts. Acquisition time was 3 min 4 s assuming 100 % navigator efficiency and heart rate of 60 beats per minute (bpm). Directly after free-breathing LGE-CMR, BH was acquired using a 3D gradient echo PSIR sequence in short-axis slice orientation in two equal stacks during two breath-holds of 10–20 s duration. Subsequently, left two-chamber and four-chamber cardiac views were acquired using the same sequence. Typical scan parameters were: TR 4.31 ms; TE 2.09 ms; TI 250–400 ms (same as free-breathing); flip angle 10°; FOV 350 × 350 mm; matrix size 188 × 125 mm; acquired pixel size 1.86 × 2.8 mm; reconstructed pixel size 1.46 × 1.46 mm; 24 slices with 10 mm thickness and – 5 mm slice gap; SENSE factor 3.

### Image analysis

MASS research software (Department of Radiology, Leiden University Medical Centre, Leiden, The Netherlands) was used for multiplanar reformatting and image analysis. BH data were analysed after offline fusion of data of the two breath-hold 3D stacks. Free-breathing 3D datasets were reformatted into two multiplanar reconstructions with identical geometry as BH: one with normal spatial resolution (FB-NR: 1.46 × 1.46 × 10 mm), matching BH, and one with high spatial resolution isotropic voxel reconstruction (FB-HR: 0.91 × 0.91 × 0.91 mm). (See Online Supplementary Material for a description of reconstruction method.) Images were analysed in random order. First, endocardial and epicardial contours were drawn manually. Second, regions with maximally hyperenhanced and hypoenhanced healthy myocardium were identified visually and a small region of interest was drawn. Third, myocardial scar was identified automatically using the validated full-width half maximum (FWHM) algorithm [2, 13, 14]. For this algorithm, maximal signal intensity (SI_max_) was defined as the mean signal intensity of the manually annotated hyperenhanced region, and minimal signal intensity (SI_min_) was defined as the mean signal intensity of the manually annotated hypoenhanced region. Myocardial scar was defined as voxels with signal intensity higher than 50 % (SI_min_ + 0.5 × (SI_max_ – SI_min_). Grey zone (only reported for ICM patients) was defined as voxels with signal intensity between 35 % (SI_min_ + 0.35 × (SI_max_ – SI_min_) and 50 % (SI_min_ + 0.5 × (SI_max_ – SI_min_). Fourth, manual adjustments to the automatically determined areas of scar were made only in cases of evident misinterpretation by the algorithm, for example in case of microvascular obstruction. Finally, scar and grey zone masses were calculated (in grams: specific weight of scar was set at 1.05 g/ml) using an in-house developed MATLAB script (Version R2012A, Natick, MA, USA).

**Fig. 1:**
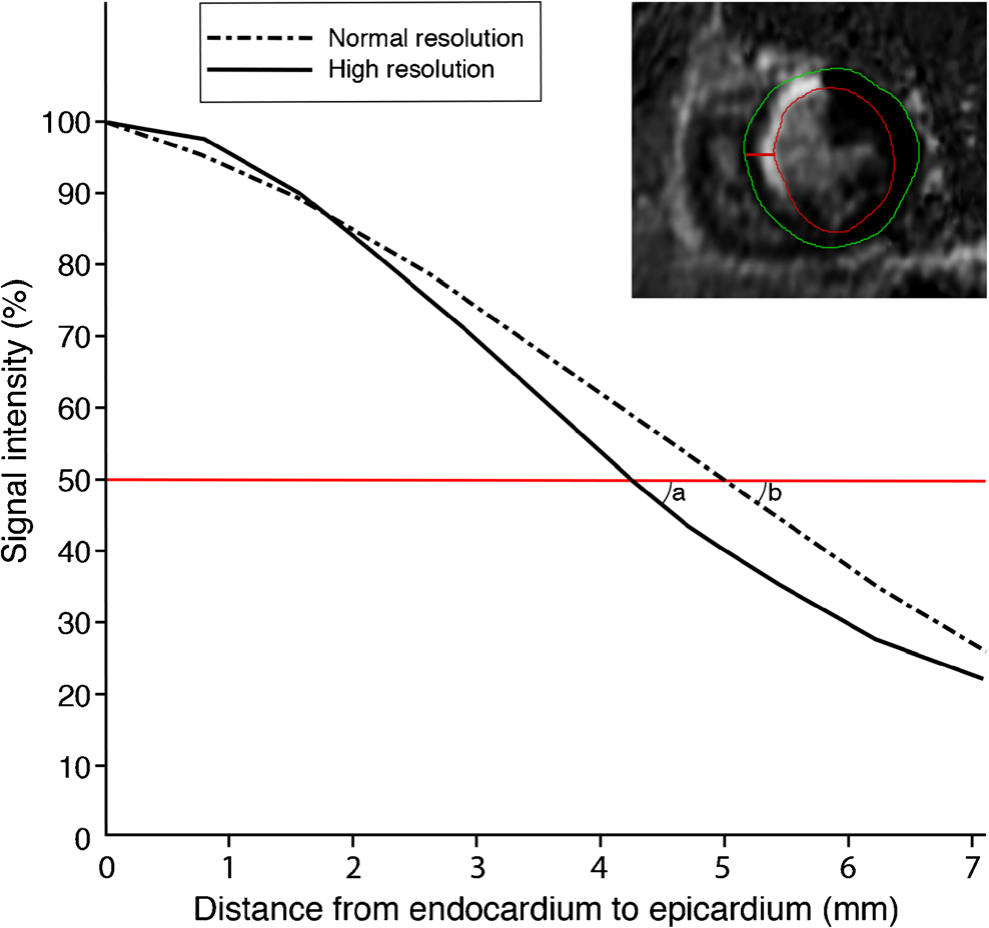
Computation of scar edge sharpness. On the upper right side of this figure a short-axis slice of a patient with anteroseptal myocardial infarction is displayed. In this slice, the red line perpendicular to the endocardial border represents the transition from dense subendocardial scar to healthy myocardium subepicardially. The graph illustrates the signal intensity alongside the red line (dotted line represents normal resolution free-breathing LGE-CMR; continuous line represents high resolution free-breathing LGE-CMR). The border of the scar is defined by the points where the red horizontal line crosses the signal intensity curves. The steepness of the slope at the scar border gives rise to the scar edge sharpness (SES), expressed in ΔSI / mm. In this figure, note that the steepness is higher in high resolution (angle *a*) as compared with normal resolution (angle *b).* It is important to appreciate that the gradient was calculated for the 3D volumes instead of the 2D representation of this figure

SNR was calculated by dividing (SI_max_ – SI_reference_) by the noise (standard deviation of signal outside the body). SI_max_ was subtracted by SI_reference_ to compensate for high signal intensity of the nulled healthy myocardium in PSIR (which is 0 in magnitude inversion recovery images) [15]. CNR was computed as the difference between SI_max_ and SI_min_, divided by the noise (standard deviation of signal outside the body). Figure 1 is a 2D representation of 3D computation of SES, which was computed with an algorithm in the Matlab environment (v. R2012a, Mathworks, Natick, MA, USA). To account for differences in signal intensity between breath-hold and free-breathing datasets, the signal intensity of all voxels containing myocardium were normalised on a scale of 0 % (SI_min_) to 100 % (SI_max_): SI_normalised_ = (SI – SI_min_) / (SI_max_ –SI_min_). Then, at the edge of the scar, the steepness of the slope (expressed in ΔSI_normalised_ / mm) reflects SES. A SES of 1.0 reflects a full transition of scar to healthy myocardium over a 1.0-mm distance.

### Statistical analysis

Data are presented as mean ± standard deviation. BH sequence was compared to FB-NR for validation purposes. Subsequently, FB-NR was compared with FB-HR. Outcome measures were compared using paired-samples *t*-test. Agreement of scar mass was compared using Pearson’s correlation coefficient and Bland Altman analysis. A *p* value of less than 0.05 was considered to indicate statistical significance. Statistical analyses were performed using SPSS software (version 20, SPSS, Chicago, IL, USA).

## Results

**Fig. 2:**
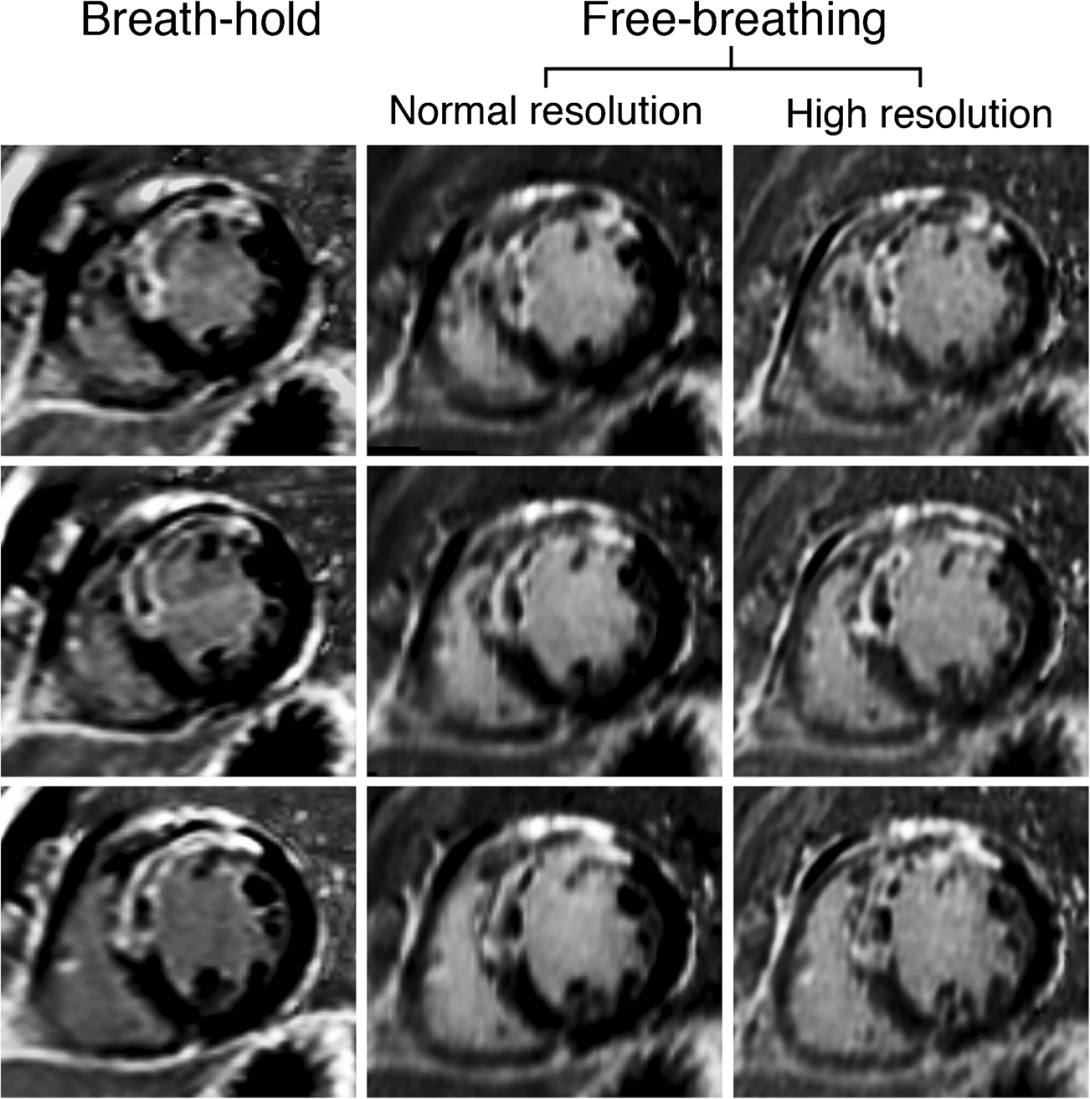
Example of patient with ischaemic cardiomyopathy. A 58-year-old man with acute myocardial infarction of the anterior wall due to obstruction in the left anterior descending artery for which primary percutaneous revascularisation was performed. Two weeks later LGE-CMR was performed. Transmural LGE of the anteroseptal wall with areas without LGE centrally in the scar can be depicted (no reflow zones). The horizontally aligned images have the exact same orientation and position in the heart and show close agreement with respect to scar visualisation. Note the sharpness of the high spatial resolution images (right panel) characterised by detailed borders of scar and areas of microvascular obstruction, as compared with normal spatial resolution (middle panel) and breath-hold (left panel) images

**Fig. 3:**
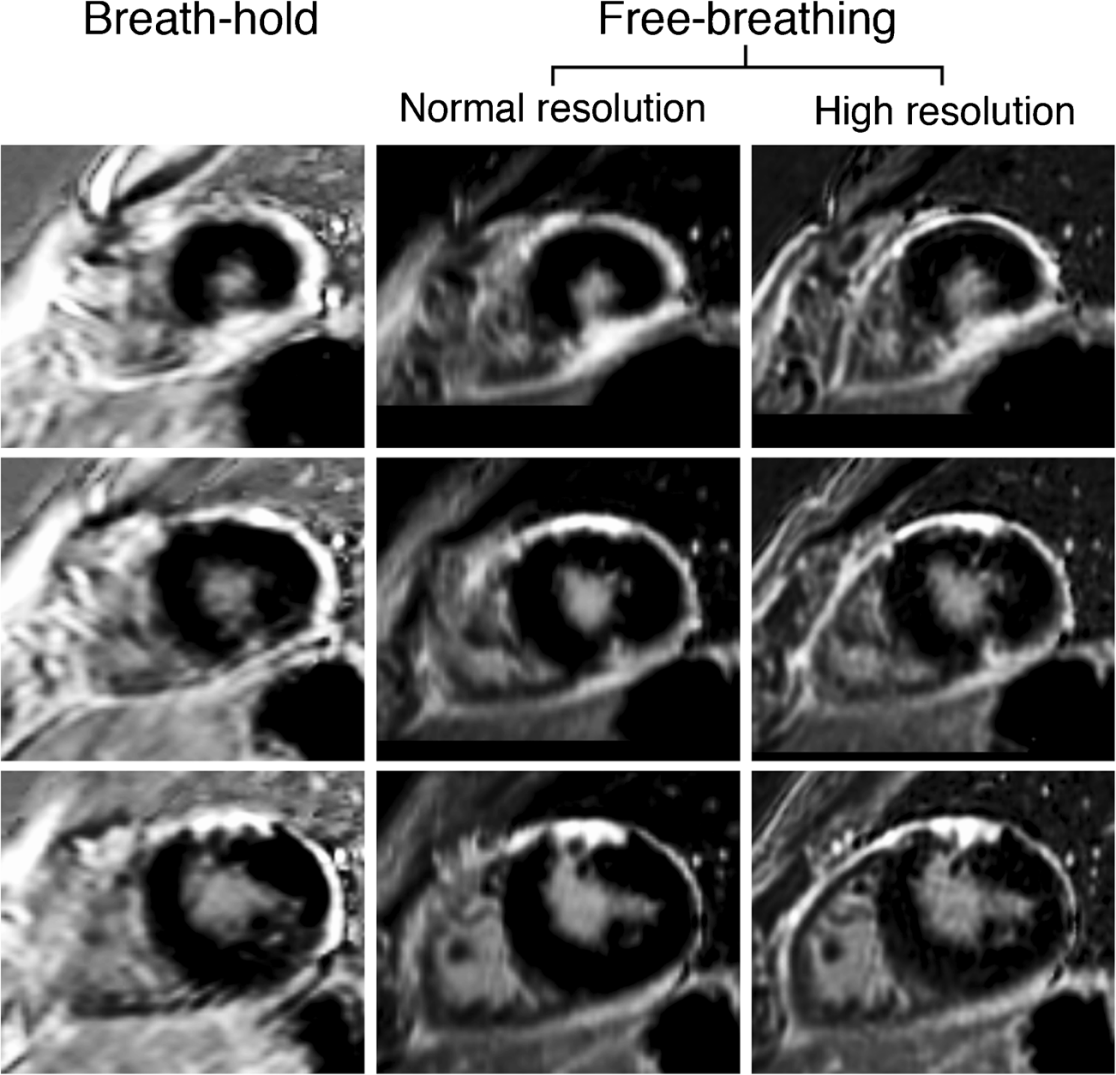
Example of a patient with perimyocarditis. A 49-year-old man who presented with thoracic pain related to breathing and posture. The electrocardiogram showed PR depression and ST elevation. CMR was performed for diagnostic purposes. LGE-CMR shows two focal subepicardial areas of enhancement (apical – mid inferior, and mid – basal anterior), as well as diffuse pericardial enhancement. It can be appreciated that the borders of scar are less blurry in the high-resolution images. Also note that the right ventricular myocardium, and surrounding pericardial enhancement can be identified best in the high-resolution images

**Fig. 4:**
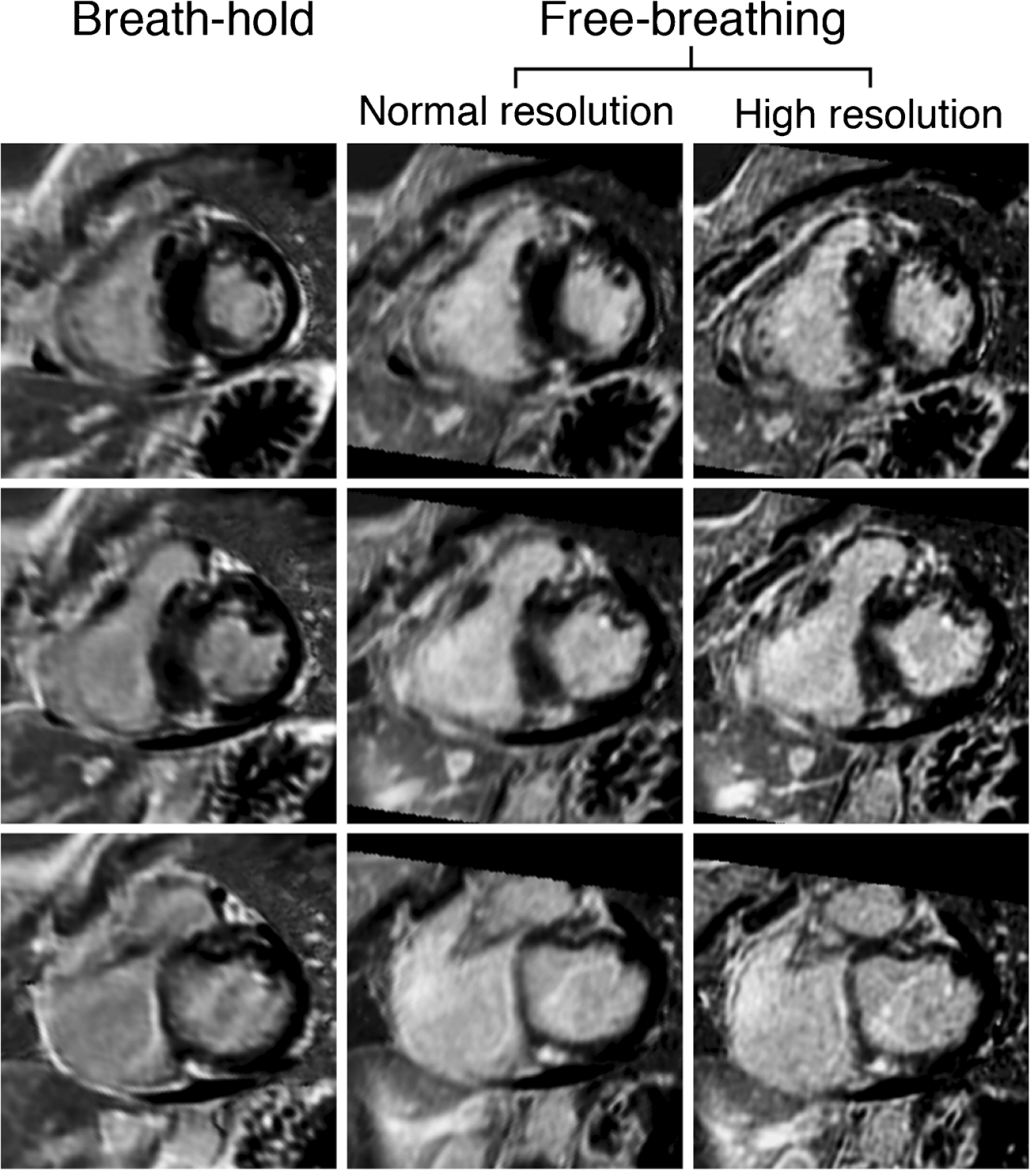
Example of a patient with hypertrophic cardiomyopathy. A 48-year-old woman with hypertrophic cardiomyopathy and non-sustained ventricular tachycardias originated from the interventricular septum. LGE-CMR images display asymmetrical septal hypertrophy and focal lesions of contrast enhancement at the right ventricular insertion point. Note close agreement of LGE area and volume in the images aligned horizontally. Furthermore, the sharpness of high spatial resolution images (right panel) is very high as reflected by the less blurry transition from scar to healthy myocardium as compared with the left and middle panel. Also note that there is less partial volume erroring of the right ventricular wall in high resolution images as compared with breath-hold and free-breathing normal resolution

**Fig. 5:**
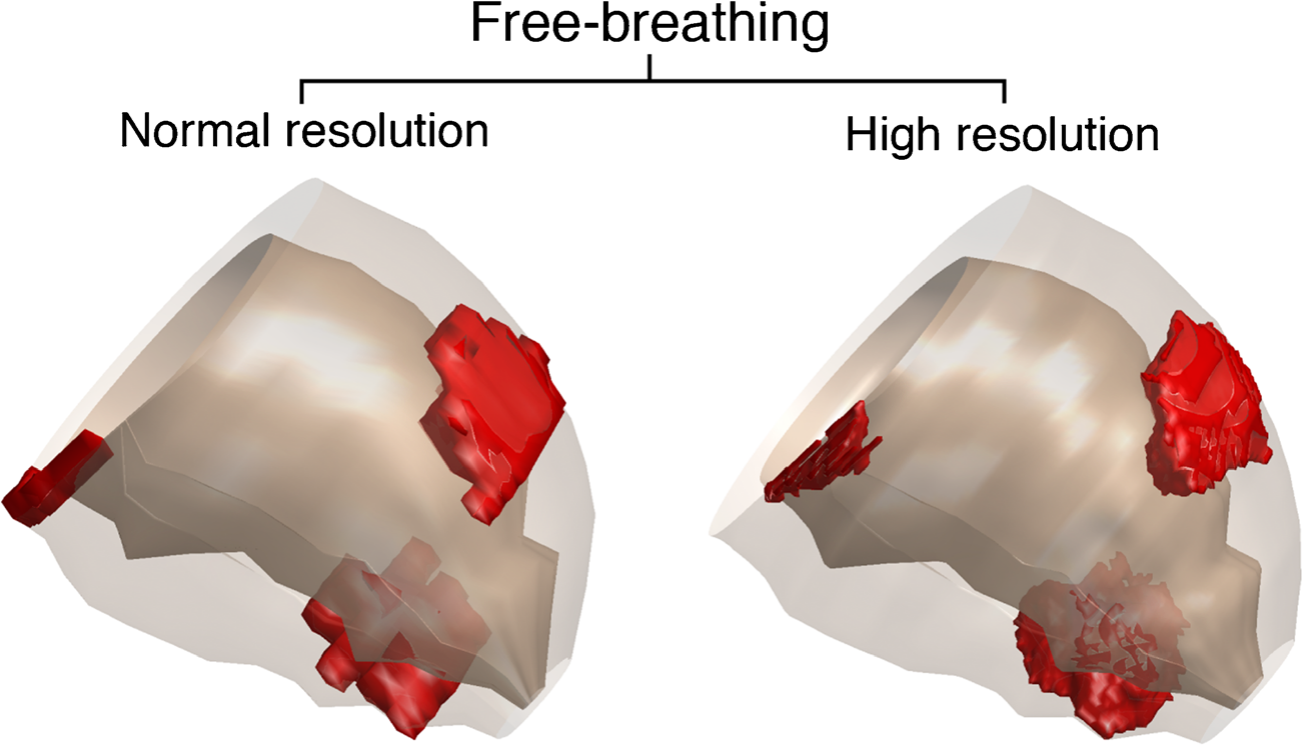
Normal versus high spatial resolution 3D reconstruction of left ventricle. Both a normal spatial resolution (left panel, 1.68 × 1.68 × 10 mm) and high spatial resolution (right panel, 0.91 × 0.91 × 0.91 mm) 3D reconstruction were generated from the free-breathing dataset of the patient represented in Fig. 3. This reconstruction shows the sharper delineated scar border in the high spatial resolution image

Of the 51 patients included, three had free-breathing images of insufficient quality due to poor ECG-triggering, breathing artefact and absence of clear myocardial scar, respectively. The remaining 48 patients were analysed (36 male; 12 female; age 60.8 years ± 10.9 years; BMI 27.6 kg/m^2^ ± 5.1 kg/m^2^), of which 34 had ICM (seven acute, four subacute, 23 chronic; 27 male; seven female; age 61.1 years ± 8.4 years; BMI 28.3 kg/m^2^ ± 5.1 kg/m^2^) and 14 had NICM (dilated cardiomyopathy n = 5; hypertrophic cardiomyopathy n = 4; acute myocarditis n = 3; other n = 2; 9 male; 5 female; age 59.5 years ± 15.6 years; BMI 24.8 kg/m^2^ ± 4.3 kg/m^2^). Mean acquisition time (± SD) was 4 min 22 s ± 1 min 25 s for BH LGE-CMR (all three cardiac views) and 9 min 34 s ± 3 min 4 s for free-breathing LGE-CMR. Figures 2, 3 and 4 show examples of BH, FB-NR and FB-HR images in patients with ICM and NICM. Figure 5 shows a normal versus high spatial resolution 3D reconstruction of the free-breathing dataset.

### Scar mass, all patients (n=48)

Scar mass was the same in BH versus FB-NR (mean ± SD: 15.5 g ± 18.0 g vs. 15.5 g ± 16.9 g, *p* = 0.997). Moreover, scar mass showed good correlation (r = 0.953) without systematic bias in Bland Altman analysis (mean difference ± SD: 0.00 g ± 5.47 g), as shown in the Online Supplementary Material. Limits of agreement were between −10.94 g and +10.93 g.

FB-NR yielded higher scar mass as compared with FB-HR (15.5 g ± 16.9 g vs. 14.4 g ± 15.6 g; *p* = 0.007). Although the data correlated well (r = 0.988), Bland Altman analysis demonstrated a systematic positive bias of + 1.15 g for FB-NR versus FB-HR (mean difference ± SD: + 1.15 g ± 2.84 g), as shown in the Online Supplementary Material. Limits of agreement were between –4.42 g and +6.72 g.

### Scar and grey zone mass ICM patients (n=34)

In the ICM subgroup, scar mass was 18.9 g ± 20.2 g in BH versus 19.3 g ± 18.7 g in FB-NR, which was not significantly different (*p* = 0.670). In parallel with scar mass in all patients, FB-NR overestimated scar mass as compared with FB-HR in the ICM subgroup (19.3 g ± 18.7 g vs. 17.9 g ± 17.2 g, *p* = 0.01).

Grey zone was not significantly different between BH versus FB-NR (6.5 g ± 5.4 g vs. 6.0 g ± 6.0 g, *p*=0.128), and between FB-NR vs. FB-HR (6.0 g ± 6.0 g vs. 6.2 g ± 6.0 g, *p*=0.329).

### Scar mass NICM patients (n=14)

In NICM patients scar mass was similar in BH vs. FB-NR (7.3 ± 6.1 g vs. 6.3 g ± 4.3 g, *p*=0.312). The difference in scar mass between FB-NR and FB-HR was not significant in this subgroup (6.3 g ± 4.3 g vs. 5.9 g ± 4.8 g, *p*=0.457).

### Image quality

As shown in the Online Supplementary Material, SNR and CNR were significantly lower in BH as compared with FB-NR (207.6 ± 85.8 vs. 1052.2 ± 877.2, *p* < 0.01 and 221.9 ± 70.9 vs. 864.9 ± 692.5, respectively). As a result of higher spatial resolution, SNR and CNR decreased significantly in FB-HR as compared with FB-NR (258.3 ± 94.9 vs. 1052.2 ± 877.2, *p* < 0.01 and 232.4 ± 91.5 vs. 864.9 ± 692.5, *p* < 0.01, respectively). In the ICM subgroup, SNR and CNR were lower in BH as compared with FB-NR (224.7 ± 87.3 vs. 1125.0 ± 1010.9, *p* < 0.01 and 240.4 ± 66.8 vs. 935.1 ± 789.1, *p* < 0.01, respectively). In FB-HR, SNR and CNR were lower as compared with FB-NR (272.9 ± 93.4 vs. 1125.0 ± 1010.9, *p* < 0.01 and 250.6 ± 89.1 vs. 935.1 ± 789.1, *p*< 0.01, respectively). Also in the NICM subgroup, SNR and CNR were lower in BH as compared with FB-NR (165.9 ± 67.9 vs. 875.5 ± 374.4, *p* < 0.01 and 177.1 ± 61.4 vs. 694.3 ± 330.3, *p* < 0.01, respectively), and lower in FB-HR as compared to FB-NR (222.8 ± 92.1 vs. 875.5 ± 374.4, *p* < 0.01 and 188.2 ± 84.5 vs. 694.3 ± 330.3, *p* < 0.01, respectively).

**Fig. 6:**
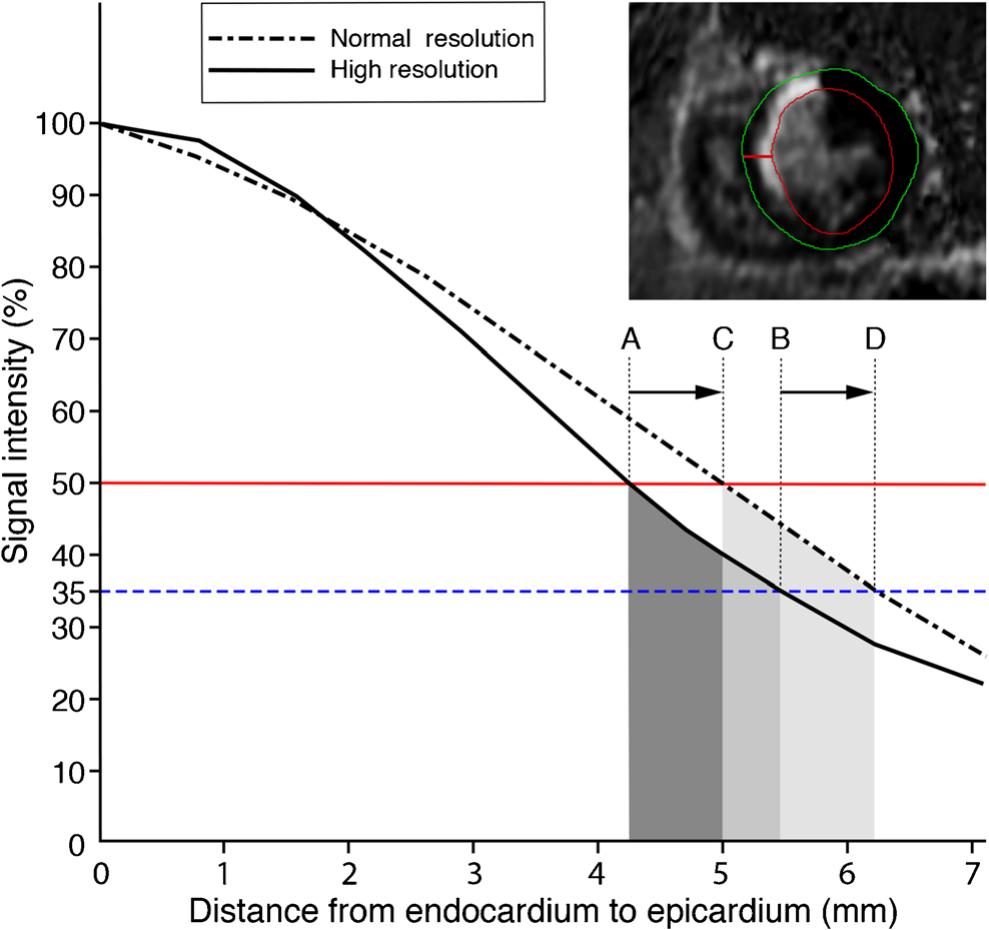
Scar and grey zone mass in relation to spatial resolution. This figure represents data of a patient with anteroseptal myocardial infarction (same patient as in Fig. 1). The signal intensity profile alongside the red line depicted in the short axis image is plotted in the graph. In the normal resolution image (dotted black line), the transition of scar to healthy myocardium is less steep as compared with high resolution (continuous line), resulting in a larger scar mass (shift from A to C). The outer grey zone border at 35 % signal intensity (horizontal blue dotted line) also shifts (A→C = B→D). Therefore, grey zone mass (illustrated by the grey areas between 35 % and 50 % signal intensity) is not larger in normal resolution (light grey area) as compared with high spatial resolution (dark grey area) LGE-CMR

SES was similar in BH versus FB-NR (0.176 ± 0.039 vs. 0.176 ± 0.036, *p* = 0.947), see Online Supplementary Material. FB-HR images had significantly higher SES as compared to FB-NR (0.216 ± 0.042 vs. 0.176 ± 0.036, *p* < 0.01). The absolute increase in sharpness was 0.040 with 95% confidence interval between 0.032 and 0.048, corresponding to a relative increase in SES of 22.7 % with 95 % confidence interval between 18.7 and 27.2. An example of the different signal intensity profiles across the edge of the scar of FB-NR vs. FB-HR is shown in Fig. 6. In the ICM subgroup, SES was the same in BH and FB-NR (0.176 ± 0.033 vs. 0.173 ± 0.036, *p* = 0.639), and statistically significantly higher in FB-HR as compared with FB-NR (0.212 ± 0.038 vs. 0.173 ± 0.036, *p* < 0.01). Also in the NICM subgroup, SES was the same in BH and FB-NR (0.176 ± 0.053 vs. 0.182 ± 0.039, *p* = 0.460), and statistically significantly higher in FB-HR as compared with FB-NR (0.227 ± 0.050 vs. 0.182 ± 0.039, *p* < 0.01).

## Discussion

This study demonstrates that, as compared with multiple breath-hold 3D LGE-CMR, navigated free-breathing 3D LGE-CMR with matched spatial resolution allows reliable scar mass quantification with good image quality. Additionally, the free-breathing technique allows to image with high spatial resolution, thereby improving scar edge sharpness and reducing scar mass. This study involves ischaemic and non-ischaemic cardiomyopathy patients in a clinical setting which emphasises that the proposed sequence could be considered an alternative for breath-hold LGE-CMR at 3T systems.

Traditionally, LGE-CMR has been performed during multiple breath-holds. Breath-holds frequently cause slice misregistration, and repeated breath-holding can be challenging for vulnerable cardiomyopathy patients [10]. This study shows that free-breathing 3D LGE-CMR yields higher SNR and CNR, as compared with breath-hold LGE-CMR with matched spatial resolution. Importantly, FB-NR allows reliable scar and grey zone mass quantification for ICM and NICM patients in a clinical setting on a 3 Tesla MR scanner. The real-life clinical setting also included vulnerable patients: population comprised 10 patients over 70 years of age, and several patients with heart failure and/or large transmural myocardial scar. Subgroup analysis of patients under and over 60 years of age revealed that there was no difference regarding image quality parameters between young and old patients (data not shown). Piehler et al. have found comparable results of motion-corrected free-breathing 2D PSIR sequence compared with a breath-hold 2D PSIR sequence with matched spatial resolution at 1.5 Tesla [10]. This study extends their findings to the 3D-PSIR acquisition technique at 3 Tesla. Schultz et al. have compared breath-hold 3D-PSIR with 3D-IR LGE-CMR at 3T showing superior image quality of the former sequence [16]. To our knowledge, this study is the first to compare a breath-hold 3D-PSIR sequence with a free-breathing 3D-PSIR sequence, with matched spatial resolution.

Because breath-holds can be challenging for patients, spatial resolution in breath-hold LGE-CMR is generally in the range of 5–10 mm in the through-plane direction. By relieving patients from repeated breath-holding, navigated free-breathing 3D LGE-CMR allows to image the whole heart without slice gaps with higher spatial resolution (especially in the through-plane direction). Our data show that, in FB-HR images, the scar border is better delineated (i.e. less blurry), as compared with FB-NR, supported by a 22.7 % relative increase in SES. Despite the increase in spatial resolution, SNR and CNR remained within the range of BH images. While others have shown subjective improvement of image sharpness of a high spatial resolution free-breathing 3D sequence against a normal spatial resolution breath-hold 2D sequence at 1.5 Tesla [17], our study is the first to objectively quantify SES in LGE-CMR. As shown in Fig. 6, we propose that increased SES in FB-HR results in smaller scar mass by reducing partial volume effects. Jablonowski et al. have shown that normal spatial resolution LGE-CMR overestimates scar mass, as compared with a high spatial resolution ex vivo sequence [11]. As such, it may be assumed that FB-HR reduces scar mass overestimation. Aforementioned study by Peters et al. found no difference in scar mass between a high spatial resolution (0.62 × 0.62 × 2.5 mm) free-breathing 3D inversion recovery sequence and a normal spatial resolution (1.2 × 1.2 × 8 mm) breath-hold 2D inversion recovery sequence in 14 ICM patients [17]. The discrepancy between our results and theirs might be explained by our larger difference in spatial resolution and different cohort size. Furthermore, we used two reconstructions from the same 3D dataset instead of two separately acquired sequences. By doing so, other variables than voxel size in itself are eliminated. Scar mass was not significantly smaller in FB-HR than FB-NR in the NICM subgroup analysis. This lack of difference is possibly related to the small sample size, and the fact that the FWHM method has lower reproducibility in NICM as compared with ICM [5].

Contrary to scar mass, grey zone mass did not differ between FB-NR and FB-HR. As illustrated in Fig. 6, the difference between the signal intensity decay of FB-NR and FB-HR diminishes at the transition area of scar to grey zone. As a result, the difference in grey zone mass is reduced to a non-significant level. It is important to note that the region characterised as grey zone probably shifts towards the endocardium in FB-HR. This suggest that FB-HR might localize grey zone more precisely. Schelbert et al. have performed *ex vivo* LGE-CMR with spatial resolutions ranging from the cellular level to approximately the level of our FB-HR reconstruction [12]. They found a positive linear correlation between grey zone mass and voxel size, explained by a reduction in partial volume averaging at the border of definite scar and healthy myocardium. However, whether their results in the *ex vivo* rat heart can be extrapolated to *in vivo* human clinical resolution images remains uncertain. Another discrepancy with our study is the method used for grey zone quantification. The FWHM method used in our study has been shown to have higher reproducibility as compared with the standard deviation threshold method used by Schelbert et al. [2, 13].

From a clinical perspective, navigated free-breathing 3D LGE-CMR has the advantage to relieve patients from repeated breath-holding and generate images with superior image quality. In theory, high spatial resolution LGE-CMR could enable more accurate visualisation of specific patterns of (very) small scar lesions, especially critical in the case of NICM. In ICM, sharper delineation of scar might better define degree of transmurality, or define the location of grey zone area, for example to improve pre-procedural guidance of ablation therapy in arrhythmia patients. Furthermore, in a research setting, scar mass quantification is often applied in (non-) ICM to correlate with adverse cardiovascular outcome. This study shows that results from LGE-CMR protocols with varying spatial resolutions should be interpreted with caution. Also, application of a high spatial resolution free-breathing 3D LGE-CMR might increase power of clinical studies that include LGE-CMR. Finally, the longer scan duration of high spatial resolution free-breathing 3D LGE-CMR must be taken into account. The scan duration of 9–10 min might be challenging for the most vulnerable or critically ill patients. In such settings, voxel size might be reduced. Alternatively, the number of slices could be reduced to include only the left ventricle instead of the whole heart. It needs to be stressed that a single free-breathing 3D LGE dataset allows reformatting in any desired plane and scan duration should be compared with acquisition of multiple 2D views.

## Limitations

Our study has several limitations. First, the reference standard of scar mass quantification is histopathological staining which was not possible to perform in this study. Second, the non-random order of sequences could have compromised SNR and CNR in breath-hold LGE-CMR (which was always performed after free-breathing LGE-CMR) due to contrast wash-out. However, both sequences were performed with PSIR compensating for suboptimal TI selection due to wash out [18, 19]. In our view, the four- to fivefold increase of SNR and CNR could not be explained by the scan order. Another limitation is the retrospective character of the study which did not allow evaluating the robustness of the proposed sequence.

## Conclusion

In a clinical setting including ischaemic and non-ischaemic cardiomyopathy patients, normal spatial resolution navigated free-breathing 3D LGE-CMR allows reliable scar mass quantification with higher SNR and CNR as compared with multiple breath-hold 3D LGE-CMR. High spatial resolution free-breathing 3D LGE-CMR has improved scar edge sharpness and reduced scar mass, as compared with normal spatial resolution free-breathing 3D LGE-CMR. High spatial resolution free-breathing 3D LGE-CMR might improve scar mass quantification and diagnostic performance, whilst relieving the patient from repeated breath-holding. In addition, free-breathing 3D LGE-CMR allows post-acquisition reformatting in any desired imaging plane.

## Electronic supplementary material

ESM 1 (DOCX 8038 kb)

## Abbreviations

BH: Breath-hold
BMI: Body mass index
CNR: Contrast-to-noise ratio
FA: Flip angle
FB-HR: High spatial resolution free-breathing
FB-NR: Normal spatial resolution free-breathing
FB: Free-breathing
FOV: Field of view
FWHM: Full width half maximum
ICM: Ischaemic cardiomyopathy
IR: Inversion recovery
LGE-CMR: Late gadolinium enhancement cardiac magnetic resonance imaging
MRI: Magnetic resonance imaging
NICM: Non-ischaemic cardiomyopathy
PSIR: Phase-sensitive inversion recovery
SD: Standard deviation
SENSE: Sensitivity encoding
SES: Scar edge sharpness
SI: Signal intensity
SNR: Signal-to-noise ratio
TE: Echo time
TI: Inversion time
TR: Repetition time
