# Supplementary material for: High spatial resolution free-breathing 3D late gadolinium enhancement cardiac magnetic resonance imaging in ischaemic and non-ischaemic cardiomyopathy: quantitative assessment of scar mass and image quality
Source: Eur Radiol. 2018 Apr 6;28(9):4027–35. doi: 10.1007/s00330-018-5361-y (PMC6096581; doi:10.1007/s00330-018-5361-y)
Supplement: Supplementary file 1 — (DOCX 8038 kb) [file 330_2018_5361_MOESM1_ESM.docx]

**Electronic Supplementary Material**


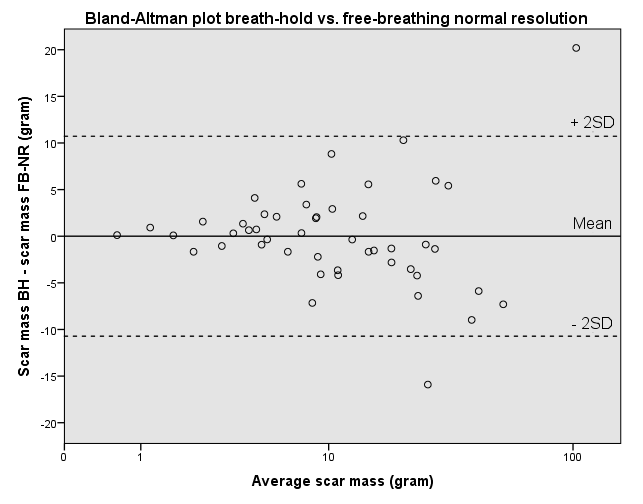


Figure 1 Bland Altman plot breath-hold LGE-CMR vs. free-breathing normal spatial resolution LGE-CMR

Agreement of scar mass between breath-hold(BH) and free-breathing sequence with matching spatial resolution (FB-NR) with average scar mass on the *x*-axis on a logarithmic scale. There is no systematic bias (mean difference = 0.0 gram). Dotted lines represent the limits of agreement (SD = standard deviation).


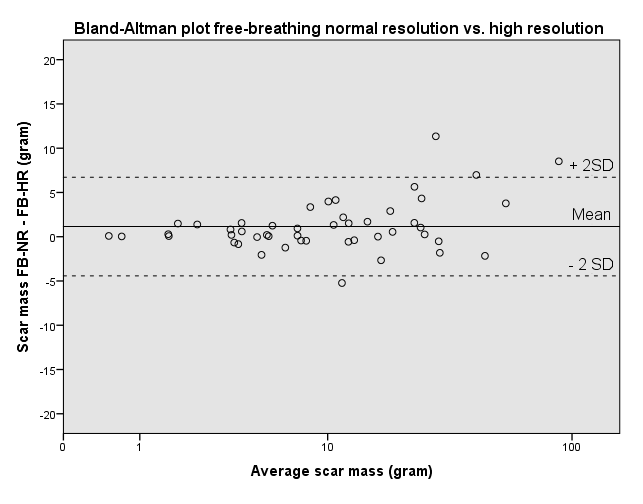


Figure 2 Bland Altman plot free-breathing normal spatial resolution LGE-CMR vs. free-breathing high spatial resolution LGE-CMR

Agreement of scar mass between free-breathing normal spatial resolution (FB-NR) and free-breathing high spatial resolution (FB-HR) LGE-CMR. The *x*-axis represents average scar mass on a logarithmic scale. There is a mean systematic bias of normal spatial resolution against high spatial resolution LGE-CMR of + 1.15 gram. Dotted lines represent the limits of agreement (SD = standard deviation).


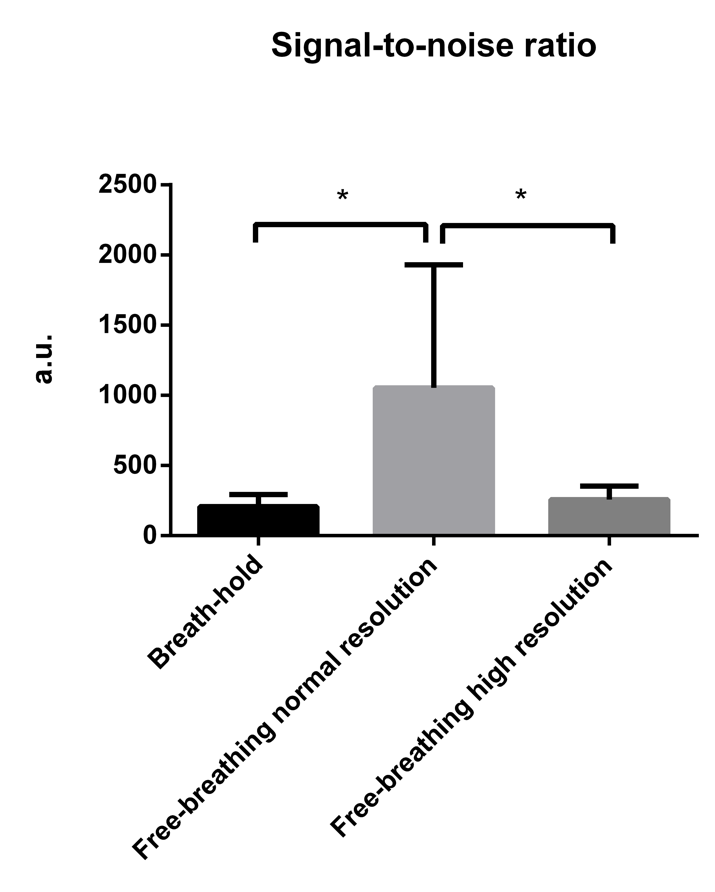


Figure 3 Signal-to-noise ratio

The signal-to-noise ratio (SNR) in arbitrary units (a.u.) for breath-holdand free-breathing normal spatial resolution and free-breathing high spatial resolutionLGE-CMR. * indicates *p* value < 0.05.


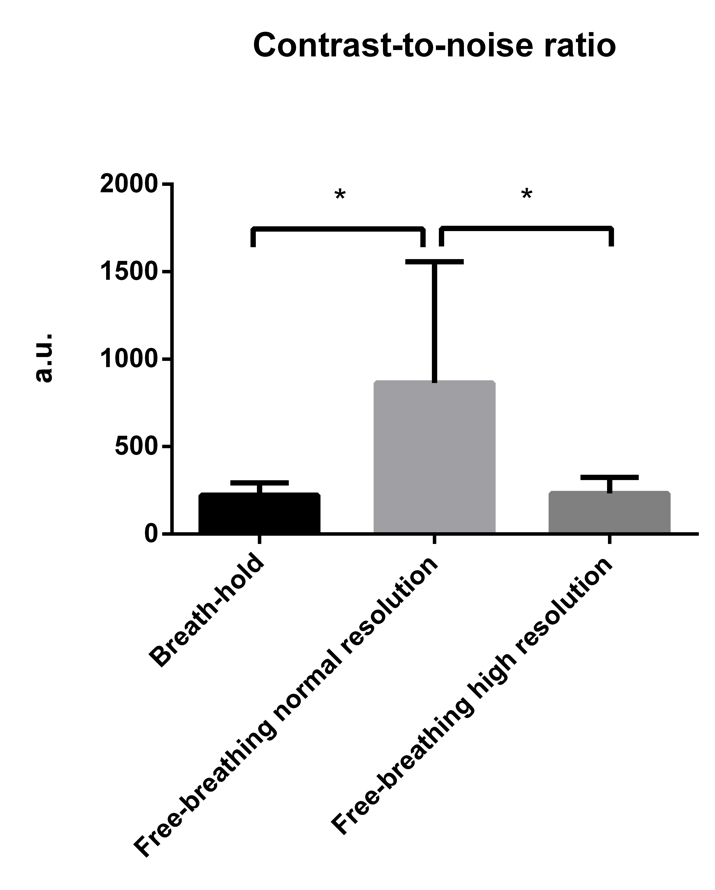


Figure 4 Contrast-to-noise ratio

The contrast-to-noise ratio (CNR) in arbitrary units (a.u.) for breath-hold, free-breathing normal spatial resolution and free-breathing high spatial resolutionLGE-CMR. * indicates *p* value < 0.05.


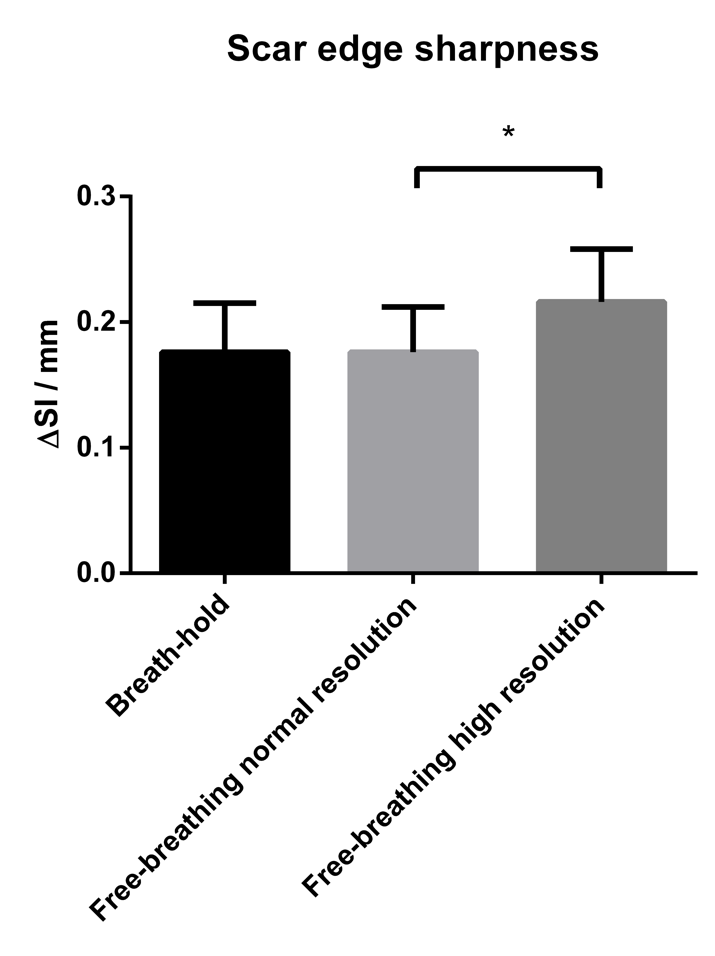


Figure 5 Scar edge sharpness

The scar edge sharpness expressed in Δ normalized signal intensity (SI) per mm for breath-hold, free-breathing normal spatial resolution and free-breathing high spatial resolution LGE-CMR. * indicates *p* value < 0.05.


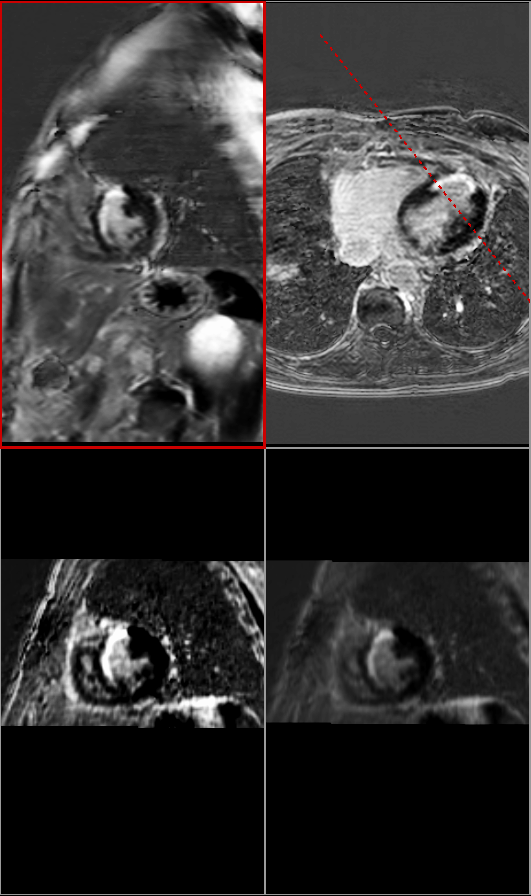


Figure 6 Multiplanar reformatting method

The BH 3D dataset was acquired in several views of which short axis (SA) view was the view for further analysis (upper left panel). Since 3D free-breathing datasets were acquired with transverse slice orientation (upper right panel), SA view needed to be reconstructed. In MASS software, geometry of BH slices was translated to the 3D FB dataset (dotted red line). As such, a SA reconstruction could be made from the 3D FB dataset. While generating such reconstruction, pixel size and slice thickness were adjusted. FB-NR was made using pixel size 1.86 x 2.8 mm and slice thickness 10 mm (lower left panel), exact to BH 3D SA slices. FB-HR was reconstructed using pixel size 0.91 mm x 0.91 mm and slice thickness 0.91 mm (lower right panel).
